# Human Deaths Related to Oleander Poisoning: A Review of the Literature

**DOI:** 10.3390/toxins17030115

**Published:** 2025-03-01

**Authors:** Matteo Antonio Sacco, Saverio Gualtieri, Aurora Princi, Alessandro Pasquale Tarallo, Maria Cristina Verrina, Lucia Tarda, Luca Calanna, Santo Gratteri, Isabella Aquila

**Affiliations:** Institute of Legal Medicine, Department of Medical and Surgical Sciences, University “Magna Graecia” of Catanzaro, 88100 Catanzaro, Italy; matteoantoniosacco@gmail.com (M.A.S.); saverio.gualtieri@studenti.unicz.it (S.G.); aurora.princi@studenti.unicz.it (A.P.); alessandropasquale.tarallo@studenti.unicz.it (A.P.T.); mariacristina.verrina@studenti.unicz.it (M.C.V.); lucia.tarda@studenti.unicz.it (L.T.); luca.calanna@studenti.unicz.it (L.C.); gratteri@unicz.it (S.G.)

**Keywords:** oleander, poisoning, death, intoxication

## Abstract

Oleander poisoning, resulting from the ingestion of Nerium oleander or Thevetia peruviana, is a serious toxicological issue in various parts of the world, particularly in regions where these plants grow abundantly and are easily accessible. Oleander contains potent cardiac glycosides, such as oleandrin and thevetin, which exert powerful effects on the cardiovascular system, leading to symptoms ranging from nausea and abdominal pain to severe arrhythmias and sudden cardiac death. This review summarizes the existing literature on the epidemiology, clinical features, pathophysiology, and challenges in treatment management associated with oleander poisoning. While supportive care, gastric decontamination, and the administration of digoxin-specific Fab antibody fragments (Digifab) are essential therapeutic measures, limited access to Digifab, delays in intervention, and insufficient supportive care practices remain significant complicating factors. Particular attention is given to findings from autopsy reports, which provide critical insights into the pathophysiological effects of oleander toxins and help bridge gaps in understanding fatal cases. This review acknowledges key limitations, particularly the scarcity of English-language publications, which restricts input from regions such as southern Asia and the Mediterranean—areas where oleander-related poisoning, especially in cases of intentional self-harm, is more prevalent. Additionally, this review highlights the socio-cultural dimensions of oleander ingestion, often linked to intentional self-poisoning, and emphasizes the need for enhanced preventive measures and public education. Future research efforts should prioritize addressing these gaps through autopsy-based studies and the development of more accessible and effective antidotes, which are essential to mitigate the global health burden of oleander-related mortality.

## 1. Introduction

### 1.1. Classification of Oleander Families

Oleander plants belong to a diverse family known for their striking beauty and potential toxicity [1]. Within the Apocynaceae family, the oleander genus comprises several species, most notably Nerium oleander and Thevetia peruviana, which are the most commonly encountered varieties [2]. These plants are widely recognized for their vibrant flowers and evergreen leaves, making them popular ornamental choices. The classification of oleander families is essential for understanding the potential risks associated with these plants, as even minor exposure can lead to serious health implications [3].

Distinguishing harmful oleanders from harmless ones involves understanding specific plant characteristics and chemical compositions [4]. Harmful oleanders, such as Nerium oleander and Thevetia peruviana, contain a cocktail of toxic substances, primarily cardiac glycosides, which are responsible for their poisonous nature [5]. These compounds can disrupt normal heart function, leading to potentially fatal outcomes if ingested [6]. Conversely, non-toxic oleander varieties lack these dangerous compounds, making them safer options for landscaping and home decoration. Recognizing these differences requires careful examination of the plant’s physical attributes and understanding the presence of toxic compounds. The leaves, flowers, and stems of harmful oleanders contain high concentrations of these toxins, making them hazardous to humans and animals alike. It is crucial for individuals, particularly those in regions where oleanders are prevalent, to be aware of these distinguishing features to prevent accidental poisoning.

### 1.2. Chemical Structure of the Toxin

Oleander (*Nerium oleander*) is one of the most toxic ornamental plants, with its dangerous effects attributed primarily to a group of cardiac glycosides, the most significant being oleandrin. Cardiac glycosides like oleandrin share a common structural framework consisting of a steroidal aglycone attached to a sugar moiety, which together determine their biological activity and pharmacokinetic properties [7]. The steroidal core of oleandrin comprises four fused rings—three six-membered rings and one five-membered ring—forming a stable structure essential for its interaction with biological targets. At the C17 position, the molecule has a lactone ring, a defining feature that facilitates its binding to the Na^+^/K^+^-ATPase enzyme [8]. The sugar portion, typically digitoxose, is connected to the steroid backbone and improves the molecule’s solubility and transport across biological membranes. This combination of structural components enables oleandrin to inhibit critical cellular functions, resulting in its potent toxic effects. The chemical properties of oleandrin make it highly lipophilic, allowing it to diffuse easily into tissues, particularly the heart, where it exerts its most dangerous effects.

### 1.3. Pharmacokinetics and Metabolism of the Toxin

The pharmacokinetics of oleandrin provide important insights into how the toxin behaves within the body following ingestion. Once consumed, oleandrin is rapidly absorbed through the gastrointestinal tract, although its bioavailability remains relatively low, estimated at around 30–40%, due to first-pass metabolism in the liver [9]. After absorption, oleandrin is widely distributed throughout the body, with a particular affinity for lipid-rich tissues such as the heart, liver, and kidneys. This high lipophilicity allows oleandrin to cross cell membranes easily and accumulate in target tissues, contributing to its prolonged toxic effects. In circulation, oleandrin binds extensively to plasma proteins, which increases its half-life and further facilitates its distribution to various organs.

Once in the liver, oleandrin undergoes metabolic processing primarily through Phase I reactions such as hydroxylation and demethylation, catalyzed by cytochrome P450 enzymes. These transformations modify the molecule to prepare it for Phase II conjugation reactions, where it is linked to glucuronic acid or sulfate groups, enhancing its water solubility for excretion [10]. The primary route of elimination is through the biliary system, with most of the metabolized toxin excreted in the feces. A smaller portion is eliminated through the kidneys in the urine. The elimination half-life of oleandrin ranges between 5 and 22 h, depending on the individual’s metabolic capacity and the dose consumed. This relatively long half-life means that oleandrin can persist in the body, prolonging its toxic effects and increasing the likelihood of severe symptoms if not managed promptly. Understanding the pharmacokinetics and metabolism of oleandrin is critical for diagnosing poisoning, determining the severity of exposure, and guiding appropriate treatment strategies.

### 1.4. Causes and Symptoms of Oleander Poisoning

Oleander poisoning often presents with cardiovascular symptoms due to the plant’s toxic cardiac glycosides, which interfere with the heart’s normal function [11]. These glycosides inhibit the Na^+^/K^+^-ATPase enzyme, leading to an increase in intracellular calcium levels and resulting in cardiac arrhythmias [12]. Patients may experience a variety of heart-related symptoms, including bradycardia, or slow heart rate, and tachycardia [11]. In severe cases, these arrhythmias can progress to more dangerous conditions such as ventricular fibrillation or cardiac arrest. The impact on the cardiovascular system is a critical aspect of oleander toxicity, necessitating immediate medical attention and intervention to prevent potentially fatal outcomes [13].

Gastrointestinal manifestations are also common in cases of oleander poisoning, often appearing soon after ingestion [14]. Symptoms typically include nausea, vomiting, and diarrhea, which result from the plant’s irritant effects on the digestive tract. These symptoms can lead to dehydration and electrolyte imbalances if not managed promptly [15]. Neurological and dermatological signs are additional indicators of oleander poisoning, although they are less common than cardiovascular and gastrointestinal symptoms [16]. Neurologically, patients may present with confusion, drowsiness, or even seizures, reflecting the plant’s effect on the nervous system. Dermatologically, contact with oleander sap can lead to skin irritation or dermatitis, particularly in sensitive individuals. These symptoms can complicate the clinical picture and pose challenges in diagnosis, especially when other causes of neurological or skin manifestations are considered. Despite the broad toxicological significance of Nerium oleander and Thevetia peruviana, this review primarily focuses on fatal poisoning cases. Understanding the mechanisms of toxicity, symptomatology, and diagnostic approaches is essential to contextualizing the forensic and clinical aspects of oleander-related deaths. Particular attention is given to the role of postmortem analysis in determining the cause of death and the epidemiological trends of fatal cases.

### 1.5. Diagnosis of Oleander Poisoning

Clinical approaches for diagnosing oleander toxicity are crucial for timely and effective treatment. When a patient presents with symptoms suggestive of oleander poisoning, such as nausea, vomiting, abdominal pain, and cardiac arrhythmias, a thorough clinical evaluation is essential [17]. Physicians should take a detailed history to ascertain possible exposure to oleander plants, particularly in regions where these plants are common [5]. Physical examination findings may include bradycardia, hypotension, and neurological symptoms such as confusion or dizziness. Immediate identification of these clinical signs can lead to prompt treatment, reducing the risk of severe complications.

Laboratory tests and biomarker identification play a vital role in confirming oleander toxicity. Blood tests can reveal elevated levels of cardiac glycosides, which are the toxic compounds found in oleander [1]. These tests are essential for differentiating oleander poisoning from other conditions with similar presentations. Digoxin therapy and the subsequent need for close clinical monitoring have led to the adaptation of turbidimetric analyses in serum biochemistry analyzers. These assays are available in nearly every hospital laboratory and cross-react with plant-derived cardiac glycosides. It has been suggested that these immunoturbidimetric assays could be used to screen for poisonings involving digoxin-like compounds, which may later be confirmed through chemical analysis [18,19].

Advanced diagnostic techniques, such as high-performance liquid chromatography, can be used to identify specific oleander toxins in the bloodstream. Additionally, electrocardiograms (ECGs) may show characteristic changes such as atrioventricular block or ventricular arrhythmias, which further support the diagnosis of oleander toxicity [20].

Differential diagnosis considerations are important to distinguish oleander poisoning from other similar toxicological emergencies. One of the primary conditions to consider is digoxin poisoning, as both oleander and digoxin affect the cardiac system in comparable ways [5]. However, specific differences exist, such as the source of exposure and the presence of other symptoms that may help clinicians differentiate between the two. Additionally, other potential causes of similar symptoms, such as electrolyte imbalances or other cardiotoxic plant ingestions, must be ruled out through comprehensive assessment and testing. This ensures accurate diagnosis and appropriate management of the patient’s condition. Also, there are numerous toxic plants containing similar cardiac glycosides, the most well known and extensively described being Digitalis. Historically, the use of digoxin as a positive inotrope led to a high incidence of poisoning. However, these cases have significantly decreased with the implementation of safer therapies for congestive heart failure. In addition to Digitalis, it is important to consider other plants containing similar cardiac glycosides and other toxic substances that may produce comparable symptoms. Examples include certain plants or honey containing grayanotoxins, compounds known for their toxic effects on the cardiovascular and nervous systems.

### 1.6. Epidemiological Data About Oleander Poisoning

A demographic analysis of affected populations revealed that certain groups are more susceptible to oleander poisoning than others. For instance, children and young adults represent a significant portion of the cases due to their curiosity and potential exposure during outdoor activities [21]. Additionally, individuals working in agriculture or gardening, who may come into contact with oleander during their daily routines, are also at risk. In some cultures, oleander is utilized for its perceived medicinal benefits, leading to intentional ingestion and subsequent poisoning incidents [22]. Socioeconomic factors also play a role, with lower-income populations potentially lacking access to proper healthcare and information about the plant’s dangers [23]. In recent years, there has been a concerted effort to raise awareness about the dangers of oleander poisoning, which has contributed to a decline in cases in certain regions. However, in areas where oleander is culturally significant or widely available, the number of incidents remains concerning. Seasonal variations also impact the trend, with some studies indicating higher poisoning rates during specific times of the year when the plant is in full bloom and more accessible to the public. Monitoring these trends is essential for public health officials to implement effective prevention and intervention measures to mitigate the impact of oleander poisoning.

### 1.7. Geographical Data About Oleander Poisoning

In regions where oleander plants are prevalent, the incidence of poisoning is notably higher. These regions are typically characterized by tropical and subtropical climates, which provide the ideal conditions for the growth of both *Nerium oleander* and *Thevetia peruviana* [6,21,22,23]. These plants are often found in abundance in parts of Asia, the Mediterranean, and certain areas of the Americas. The widespread growth of these plants in such regions increases the likelihood of accidental ingestion by both humans and animals. Additionally, oleander’s ornamental value means it is frequently planted in gardens and public spaces, further elevating the risk of exposure [6]. As a result, awareness and preventive measures are crucial in these areas to mitigate the risk of poisoning.

In contrast, in regions where oleander is less common, the incidence rates are relatively lower, often linked to accidental ingestion or misuse of the plant’s parts. However, in parts of South Asia, particularly India and Sri Lanka, oleander seeds are frequently used in intentional poisonings, such as suicides. Studies indicate that while the lethality of oleander poisoning is significant, it is often not as immediate or effective as intended, leading to prolonged suffering, severe complications, and substantial healthcare burdens [14,16]. The costs—both in terms of healthcare resources and human suffering—are particularly pronounced in regions with limited access to medical care.

In India, for instance, oleander is commonly used in traditional medicine, leading to numerous cases of accidental poisoning due to misidentification or incorrect dosage [12]. Similarly, in the Mediterranean region, oleander is a familiar sight in public gardens and along roadsides, making it a frequent source of toxicological emergencies [8]. In the United States, particularly in states like California, Arizona, and Texas, oleander is commonly cultivated as an ornamental plant. While cases of human poisoning from direct contact with local oleander plants are relatively rare due to the plant’s bitter taste deterring accidental ingestion, there have been instances of livestock poisoning when animals consume the plant material. Additionally, there is a notable risk associated with herbal supplements imported across the U.S. border that are adulterated with toxic yellow oleander (Thevetia peruviana), leading to human poisonings. These concerns highlight the importance of public education regarding the potential dangers of oleander and the need for the proper labeling of both plants and herbal products to prevent accidental ingestion and ensure safety [15,16,17,18,19,20,21,22,23,24].

The influence of local flora on oleander poisoning incidents cannot be overlooked. In regions where oleander plants are common, they often coexist with other toxic flora, which can complicate the identification and treatment of poisoning cases [12]. For example, in the southwestern United States, oleander is part of a diverse ecosystem that includes other hazardous plants, increasing the risk of mixed toxicological exposures. Moreover, in rural regions of South Asia, where oleander poisoning is often intentional, delayed access to emergency medical care further exacerbates mortality and morbidity rates [14]. Consequently, understanding the interaction between oleander and surrounding vegetation, as well as addressing healthcare access and mental health resources, is essential for developing effective prevention and response strategies in these high-risk areas.

### 1.8. Oleander Poisoning and Related Pathologies

In addition to cardiovascular effects, oleander poisoning can impact the renal and hepatic systems; however, these changes are typically secondary to cardiovascular instability rather than a direct toxic effect. Acute renal impairment, which may manifest as acute kidney injury, often results from compromised cardiac output and subsequent hypoperfusion [25,26]. Similarly, hepatic stress and elevated liver enzymes are observed due to ischemic changes and systemic hypoxia, as the liver plays a central role in managing the body’s metabolic response to poisoning.

Oleander poisoning also poses significant risks when interacting with pre-existing chronic diseases, exacerbating their severity and complicating management strategies. For individuals with chronic cardiovascular conditions, the introduction of cardiac glycosides from oleander can precipitate acute decompensations, further destabilizing their condition [1]. Similarly, those with chronic renal impairment may experience a rapid decline in kidney function due to the additional toxic load. This interaction underscores the complexity of managing oleander poisoning in patients with underlying health issues, highlighting the importance of a tailored approach to treatment that considers both the acute toxicological impact and the chronic disease context.

### 1.9. Oleander Poisoning and Death

Despite the dangerous potential of oleander ingestion, the human mortality rate is generally low, even in cases of intentional consumption, such as suicide attempts [27]. Case reports of fatal incidents highlight the deadly nature of oleander poisoning. In South Asia, for instance, there are tens of thousands of yellow oleander poisoning cases each year, resulting in probably thousands of deaths [12].

Several factors influence the lethality and prognosis of oleander poisoning. The toxic effects are primarily due to the ingestion of multiple leaves or large doses of the plant’s components, such as decoctions [12]. The severity of poisoning can vary depending on the amount ingested, the individual’s health status, and the promptness of medical intervention. Geography and seasonality also play roles, as oleander exposure is a year-round risk, particularly in certain U.S. states like California, Arizona, and Texas, where the plant is prevalent [26]. While non-fatal cases of oleander poisoning are frequently documented, fatal outcomes remain a critical concern. The ability to accurately determine the cause of death in these cases relies heavily on postmortem examinations, including histopathological and toxicological analyses. This review highlights the role of forensic pathology in identifying oleander-related fatalities and the challenges in distinguishing these cases from other causes of sudden cardiac death.

## 2. Results

Several recent studies have examined the epidemiology, clinical presentation, biochemical effects, autopsy findings, and management strategies for oleander poisoning, which remains a significant cause of morbidity and mortality in many parts of the world.

A retrospective study by Krishnasamy et al. analyzed 2123 poisoning cases in a tertiary care hospital in South India and found that oleander poisoning accounted for a significant proportion of admissions, particularly among young, rural males [28]. Most cases were linked to deliberate self-harm, and the Glasgow Coma Scale (GCS) at admission was identified as a strong predictor of mortality. Lower GCS scores were associated with poorer outcomes, emphasizing the need for rapid identification and intervention. Similarly, Ayyappan et al. reported a pediatric case of accidental ingestion of *Nerium oleander* leaves [29]. The child exhibited severe symptoms such as vomiting, hypotension, hyperkalemia, and myocardial dysfunction, ultimately leading to death within 36 h. The autopsy findings revealed widespread petechial hemorrhages, centrilobular necrosis in the liver, and acute tubular necrosis in the kidneys, underscoring the systemic effects of oleander toxins.

In a regional analysis by Torrents et al. conducted over 20 years in Southeastern France, deliberate oleander ingestion was the leading cause of plant-related poisonings, accounting for 71% of reported cases [30]. The study documented four fatalities, with autopsies revealing multi-organ congestion and cardiac failure. Despite the use of Digifab (digoxin-specific Fab fragments) in severe cases, the study noted the lack of standardized treatment protocols as a significant limitation in managing oleander toxicity. This highlights the need for consistent therapeutic approaches and increased clinical awareness.

A detailed observational study by Subramani et al. focused on cases of *yellow oleander* poisoning in India [1]. Clinical findings included persistent vomiting, abdominal pain, dizziness, and severe hyperkalemia, often accompanied by electrocardiographic abnormalities such as bradycardia, atrioventricular (AV) blocks, and ST-segment changes. The study found that the ingestion of crushed seeds caused more severe cardiac manifestations compared to whole seeds, with serum potassium levels serving as a critical predictor of cardiotoxicity and patient outcomes [1]. These findings emphasize the importance of close electrolyte monitoring and early intervention in the management of oleander poisoning.

From a forensic perspective, Carfora et al. described a unique suicide case involving the deliberate ingestion of a *Nerium oleander* infusion [31]. Toxicological analysis confirmed high concentrations of oleandrin in multiple biological samples, with fatal cardiac arrhythmia identified as the cause of death. Similarly, Papi et al. reported a rare case of double fatalities due to the accidental ingestion of oleander leaves, where toxicological findings confirmed the presence of cardiac glycosides [32]. These studies underscore the importance of advanced toxicological methods like LC-MS/MS for the accurate identification of oleander toxins and their metabolites in clinical and forensic settings.

Autopsy findings have proven invaluable in elucidating the systemic effects of oleander poisoning. In addition to cardiac failure, postmortem examinations have frequently revealed acute renal failure, multi-organ congestion, and myocardial dysfunction, as documented in multiple studies. Nevertheless, autopsies remain critical tools in correlating the ingestion of oleander with the observed clinical and biochemical effects, as well as providing forensic evidence in cases of intentional or accidental ingestion.

The management of oleander poisoning remains particularly challenging in resource-limited settings. Roberts et al. reviewed treatment options for cardiac glycoside poisoning, emphasizing the efficacy of interventions such as activated charcoal, atropine, and temporary cardiac pacing [33]. However, the use of digoxin-specific Fab fragments, the most effective antidote, is often limited by financial and logistical constraints. This underscores the urgent need for accessible and affordable therapeutic interventions.

Bandara et al. provided a comprehensive review on the diagnosis and clinical management of poisoning caused by *Nerium oleander* and *Thevetia peruviana* [15]. The authors highlighted the primary clinical manifestations, including gastrointestinal symptoms, dysrhythmias, and hyperkalemia [15]. Their findings emphasized the importance of supportive care, activated charcoal, and the administration of digoxin-specific Fab fragments as key strategies for managing severe toxicity.

Expanding on treatment options, Gawarammana et al. conducted a randomized controlled trial investigating the efficacy of *Fructose 1,6-diphosphate* (FDP) as a novel antidote for yellow oleander-induced cardiac toxicity [34]. FDP showed potential benefits in reversing life-threatening arrhythmias, providing an inexpensive and practical alternative in resource-limited settings where digoxin-specific Fab is often unavailable. This study demonstrated encouraging results, suggesting that FDP could be a life-saving intervention.

Carroll et al. explored the relationship between the time of ingestion and patient outcomes in oleander poisoning [35]. Their epidemiological study revealed strong evidence of chronotoxicity, with a significantly lower case fatality rate observed in patients who ingested oleander in the evening compared to other times of the day. This novel finding suggests that the timing of exposure may influence toxicity, likely due to variations in metabolism or physiological responses throughout the day.

Finally, Anandhi et al. reported a rare case of acute myocardial infarction following yellow oleander poisoning [11]. While cardiac dysrhythmias are commonly documented in oleander toxicity, this study presented a case where the patient initially displayed a normal sinus rhythm but developed acute ST-segment elevation myocardial infarction within hours of ingestion. This finding underscores the wide spectrum of cardiac complications that can occur in oleander poisoning and highlights the need for vigilant cardiac monitoring.

Similarly, the study by Bose et al. documents 300 cases of oleander poisoning, of which only 14 resulted in fatalities [36].

In summary, the reviewed studies collectively demonstrate the global burden of oleander poisoning and its severe cardiac and systemic toxicity. Whether accidental or intentional, the ingestion of oleander seeds or leaves can rapidly lead to fatal outcomes due to the potent effects of its cardiac glycosides. Autopsy findings play a critical role in identifying the cause of death, often revealing multi-organ damage and myocardial dysfunction. The importance of early diagnosis, close monitoring of electrolyte imbalances, and access to specific antidotes cannot be overstated. While significant progress has been made in understanding the toxicology and clinical presentation of oleander poisoning, gaps remain in standardized treatment protocols and antidote availability, particularly in low-resource settings. Future research efforts must focus on improving therapeutic strategies, raising public awareness, and implementing preventive measures to reduce the incidence of oleander poisoning worldwide (Table 1).

## 3. Discussion

### 3.1. Detection Methods

Detecting oleander poisoning involves identifying and quantifying oleandrin in biological samples such as blood, urine, or tissues using sensitive analytical methods. Among these, high-performance liquid chromatography coupled with mass spectrometry (HPLC-MS/MS) is the most reliable and widely used technique [7,37].

HPLC-MS/MS works by first extracting oleandrin from a complex biological matrix using solvent-based methods, then separating it through a chromatographic column under high pressure. Once separated, the compound is analyzed based on its mass-to-charge ratio, allowing for precise identification even at extremely low concentrations. This method is highly sensitive, specific, and capable of detecting oleandrin concentrations below one nanogram per milliliter, making it invaluable in forensic and clinical toxicology.

Gas chromatography–mass spectrometry (GC-MS) is another powerful detection technique often used to analyze oleandrin, particularly in cases where the compound has been chemically derivatized to increase its volatility for separation [38]. GC-MS provides excellent resolution and is particularly useful for detecting oleandrin metabolites in biological fluids [38]. In emergency or clinical settings where rapid screening is necessary, immunoassays such as ELISA (Enzyme-Linked Immunosorbent Assay) are commonly used. ELISA relies on antibodies specific to oleandrin, which bind to the toxin and generate a measurable signal. While immunoassays are faster and simpler than chromatographic methods, they are less specific and may cross-react with structurally similar compounds. For laboratories with limited resources, Thin-Layer Chromatography (TLC) offers a qualitative detection method. In TLC, oleandrin is separated on a silica gel plate using specific solvents and visualized under UV light or chemical staining. Although less sensitive than other methods, TLC can confirm oleandrin’s presence and support the diagnosis of poisoning in less advanced settings.

The detection of oleander poisoning involves identifying and quantifying cardiac glycosides, primarily oleandrin, in biological samples such as blood, urine, or tissues. Advanced analytical techniques like ultra-high-performance liquid chromatography coupled with tandem mass spectrometry (UHPLC-MS/MS) are employed for this purpose. This method has demonstrated a limit of detection (LOD) of 0.11 ng/mL and a limit of quantitation (LOQ) of 0.36 ng/mL in blood samples [37]. 

The toxicity of oleander is significant, with reports indicating that the ingestion of approximately 4 g of oleander leaf can be lethal to an adult [39]. In one documented case, a blood oleandrin concentration of 1.1 ng/mL was associated with severe poisoning symptoms, including nausea, vomiting, abdominal pain, cardiovascular shock, and sinus bradycardia [40]. These findings underscore the plant’s high toxicity and the importance of prompt medical attention following ingestion.

### 3.2. Oleander Poisoning and Death

Fatalities associated with oleander poisoning remain a significant concern. While many cases of Nerium oleander and Thevetia peruviana poisoning do not result in death, severe toxicity leading to fatal outcomes is most commonly observed in cases of intentional ingestion or in patients with pre-existing cardiovascular conditions. A retrospective study in South Asia reported that, among tens of thousands of yellow oleander poisoning cases annually, approximately 1000 result in fatalities [12]. Another epidemiological review found that the mortality rate among hospitalized oleander poisoning cases ranges from 2% to 10%, with significantly higher fatality rates in rural areas with limited treatment options [14]. In a cohort study of 300 oleander poisoning cases, 14 fatalities were recorded, emphasizing that while severe toxicity is common, survival is achievable with appropriate medical intervention [36]. Similarly, a 20-year retrospective analysis in France documented 71% of plant-related poisonings involving oleander, with four confirmed deaths, further underscoring the importance of early and effective treatment [29].

Forensic and clinical reports indicate that intentional ingestion accounts for the majority of fatal cases [28]. Studies from India and Sri Lanka reveal that young rural males are particularly affected, with oleander seeds or leaves frequently used in cases of deliberate self-poisoning [1,28]. In contrast, accidental pediatric poisonings, though relatively common, rarely lead to death due to smaller ingested doses and prompt medical intervention [29]. A pediatric case report described a child who ingested oleander leaves and succumbed within 36 h, with autopsy findings indicating severe myocardial damage, acute renal failure, and centrilobular hepatic necrosis [29].

The geographic distribution of fatal oleander poisoning cases follows the plant’s natural habitat. The highest incidence is reported in South Asia, the Mediterranean, and parts of Africa, where oleander is abundant and often used in traditional medicine [12]. However, fatalities have also been documented in the United States, particularly in California, Arizona, and Texas, where both intentional and accidental poisonings have been reported [25,26,27].

The severity of oleander poisoning is influenced by the ingested dose, mode of ingestion, and patient-specific factors. Studies indicate that consuming more than 15–20 Nerium oleander leaves can be fatal in adults [12], while the ingestion of 5–10 crushed Thevetia peruviana seeds carries a significant risk of mortality [1]. Oleander tea or infusions pose an even higher risk due to the concentrated extraction of cardiac glycosides, as observed in forensic cases of deliberate ingestion [17]. Patient-specific factors such as pre-existing cardiac conditions further increase the likelihood of fatal arrhythmias [1]. Delayed medical intervention beyond six hours post ingestion has been associated with a higher risk of mortality [35], and severe hyperkalemia (>6.5 mmol/L) has been identified as a strong predictor of fatality [1]. A novel study suggested that chronotoxicity might influence mortality, with ingestions occurring in the evening associated with lower fatality rates, possibly due to circadian variations in metabolism [35].

The clinical progression of fatal oleander poisoning follows a distinct pattern. Initial gastrointestinal symptoms, including nausea, vomiting, and diarrhea, appear within two hours of ingestion. These are followed by cardiac dysrhythmias, hyperkalemia, and progressive central nervous system depression within 12–24 h. If untreated, patients typically succumb to cardiac arrest within 24–48 h. A study by Ayyappan et al. reported a fatal case where a patient developed profound hypotension and myocardial dysfunction, leading to death within 36 h [29]. Another case series indicated that patients who survived beyond 48 h had a significantly higher probability of full recovery [1].

Common postmortem findings include myocardial congestion, necrosis, and histopathological evidence of direct cardiotoxic effects [41]. Additional systemic effects include acute tubular necrosis and centrilobular hepatic necrosis, often secondary to ischemic injury [41]. Severe hyperkalemia is consistently documented in postmortem blood samples, frequently exceeding 7.0 mmol/L in fatal cases [1]. Advanced toxicological analysis, particularly liquid chromatography–mass spectrometry (LC-MS/MS), plays a critical role in detecting oleandrin and related cardiac glycosides in biological samples [42].

Oleander poisoning presents significant forensic and clinical challenges. While the majority of poisonings are non-fatal, mortality is primarily observed in cases of intentional ingestion, delayed medical intervention, and severe hyperkalemia. Forensic pathology and toxicology remain crucial in identifying oleander-related deaths, yet gaps persist in the standardization of postmortem toxicological analyses and epidemiological data collection. Future research should focus on improving access to specific antidotes, standardizing forensic toxicology protocols, and developing preventive strategies to mitigate the global burden of oleander poisoning.

### 3.3. Literature Review of Oleander Poisoning Cases

The medical literature documents numerous cases of oleander poisoning, each providing valuable insights into the clinical presentation and outcomes of such incidents. Historically, oleander poisoning incidents have predominantly been accidental, with intentional ingestion being relatively rare [4]. This aligns with the broader trend of poisoning cases, where accidental exposure often overshadows deliberate self-harm, especially in regions where oleander grows naturally and is used for medicinal purposes [43]. The historical context is further enriched by oleander’s use in traditional medicine across various cultures, particularly in the Indian subcontinent and Turkey, where it has been employed for treating ailments like cancer and hyperglycemia [31]. While this historical usage underscores the plant’s significant role in traditional medicine, it also raises concerns about the potential for accidental poisoning due to its widespread availability and potent toxicity.

In recent decades, there has been a noticeable trend in the reporting of oleander poisoning cases, reflecting shifts in public awareness, medical diagnostics, and reporting accuracy. The apparent increase in documented cases may be attributed to improved diagnostic techniques and heightened awareness of oleander’s toxic potential among medical professionals and the general public [29]. This trend does not necessarily signify a true rise in poisoning incidents but rather reflects enhanced recognition and reporting capabilities. Additionally, oleander plant populations in tropical and subtropical regions, where the plant thrives, appear largely unchanged, suggesting that environmental factors are not the primary drivers of this trend.

Despite the growing number of reported cases, the number of autopsies conducted in fatal incidents remains disproportionately low. This gap highlights the need for more comprehensive postmortem analyses to better understand the full range of physiological effects caused by oleander toxins. Such analyses are critical for improving diagnostic precision, identifying subtle pathological changes, and facilitating the development of targeted treatments and antidotes. Bridging this gap in forensic investigations could significantly enhance clinical and preventive strategies, ultimately improving outcomes for oleander poisoning cases.

### 3.4. Autopsy Procedures and Data in Oleander Poisoning Cases

Conducting an autopsy in cases of suspected oleander poisoning requires a meticulous and systematic approach to uncover the cause of death. The process begins with a thorough external examination, focusing on identifying any visible signs of oleander ingestion, such as remnants of plant matter around the mouth or lips. Internally, particular attention is given to the gastrointestinal tract, where plant material in the gastric contents can provide valuable evidence. Oleander leaves, for instance, have a characteristic parallel vein pattern, which can be confirmed microscopically, as demonstrated in documented cases [44]. For more definitive confirmation, DNA barcoding techniques can identify plant material at a molecular level, offering robust proof of exposure [45].

The pathologist closely examines the heart, as oleander toxins are known to cause functional and structural cardiac damage. Functional damage, such as arrhythmias and conduction abnormalities, often occurs early and may precede visible structural lesions. However, postmortem and microscopic examination can reveal myocardial lesions that serve as key indicators of oleander poisoning. Myocardial necrosis, fibrosis, and myocarditis are considered characteristic findings but may present inconsistently [29]. If the fatal dose is large and sudden, myocardial cells may only show subtle swelling and hypereosinophilia, reflecting acute toxic effects. In contrast, lower doses over longer durations are more likely to produce necrosis, subsequent fibrosis, and inflammation [46,47,48,49,50,51,52]. These findings highlight the variability in myocardial pathology depending on the dose, exposure duration, and survival time.

In addition to cardiac findings, biochemical markers such as troponin and CPK can provide further evidence of myocardial injury, supporting the pathological observations. Toxicological analysis of blood, urine, and tissue samples is essential for confirming the presence of cardiac glycosides. These compounds, found in *Nerium oleander* and *Thevetia peruviana*, can be identified and quantified using advanced analytical techniques such as liquid chromatography–mass spectrometry (LC-MS). Rapid screening methods, including digoxin immunoassays, can also detect oleander toxins due to their cross-reactivity with digoxin antibodies [45,46]. Combining biochemical, toxicological, and pathological findings enhances the reliability of confirming oleander poisoning as the cause of death.

Secondary organ involvement, including renal and hepatic changes, may also be observed and is typically secondary to cardiovascular compromise. Reduced perfusion, systemic hypoxia, and electrolyte imbalances can result in elevated liver enzymes and signs of acute kidney injury. The inclusion of myocardial lesions—whether subtle swelling and hypereosinophilia in acute cases or necrosis with later fibrosis in prolonged exposures—adds valuable context to the clinical picture. Incorporating evidence from toxicological analyses and advanced identification techniques like microscopy, DNA barcoding, and LC-MS enhances the accuracy and comprehensiveness of the findings.

These autopsy reports serve multiple critical purposes. They provide the deceased’s family with a clear understanding of the cause of death, support public health initiatives by highlighting the risks associated with oleander exposure, and contribute to legal investigations where poisoning is suspected.

Statistical analysis of autopsy findings in oleander poisoning cases provides critical insights into the frequency, nature, and outcomes of these incidents. Both accidental and intentional ingestions of oleander contribute significantly to cases requiring postmortem examination [48]. The primary cause of death in such cases is often cardiac arrhythmia, which reflects the profound impact of oleander toxins on the heart. Postmortem examinations frequently reveal specific cardiac abnormalities that can lead to bradycardia and various forms of atrioventricular (AV) block, consistent with the toxicological effects of cardiac glycosides [33].

### 3.5. Discrepancy Between Reported Deaths and Autopsy Cases

The small number of autopsies conducted compared to the reported deaths from oleander poisoning presents a significant gap in understanding the full impact of this toxic plant. The rarity of autopsies in such cases may stem from a perception that the overt symptoms and clinical history provide sufficient evidence for determining the cause of death. However, without detailed autopsy results, the specific physiological mechanisms leading to death remain inadequately explored [49]. Additionally, resource constraints, including lack of funding and trained personnel, may impede the ability to perform autopsies in every suspected poisoning case. Another factor could be the legal and administrative challenges that sometimes accompany the autopsy process, discouraging its routine application. Collectively, these reasons point to an urgent need for raising awareness and improving resources to ensure that autopsies are more routinely conducted in oleander poisoning cases.

The discrepancy between reported deaths and autopsy cases of oleander poisoning has significant implications for medical research and public health policies. This gap limits the ability of researchers to accurately determine the true incidence and mortality rates associated with oleander poisoning, leading to potential underestimation of its public health impact [5]. Without robust data from autopsies, it becomes challenging to develop targeted antidotes or clinical interventions that could mitigate the effects of the poison. Furthermore, the lack of detailed toxicological data hinders the formulation of effective public health strategies aimed at prevention and education. Addressing this discrepancy is crucial for advancing scientific knowledge and improving health outcomes related to oleander poisoning.

### 3.6. Insights from Animal Studies and Their Relevance to Human Toxicology

Experimental and veterinary clinical studies have significantly contributed to understanding the toxic effects of oleander and have provided insights that complement human clinical findings. While Nerium oleander and Thevetia peruviana poisonings primarily affect humans, numerous cases have been reported in livestock, companion animals, and experimental models, allowing for comparative toxicological analysis. A study by Sykes et al. investigated renal lesions in horses affected by oleander poisoning and documented tubular necrosis, hyaline casts, and vascular congestion, suggesting that renal impairment in oleander toxicity is likely secondary to systemic hypoperfusion rather than direct nephrotoxicity [40]. This aligns with human autopsy findings where acute tubular necrosis is often observed but is not specific to oleander poisoning. Similarly, Roberts et al. reviewed treatment strategies in veterinary medicine and emphasized the use of activated charcoal, atropine, and fluid resuscitation while noting the variable efficacy of digoxin-specific Fab fragments (Digifab) in counteracting toxicity across different species [33].

Another relevant study by Fattahi et al. examined the potential protective effects of garlic extract in a sheep model of oleander poisoning [50]. Their research demonstrated a significant reduction in mortality and the delayed onset of cardiac arrhythmias, suggesting that garlic-derived compounds may modulate ion channel function and counteract cardiac glycoside effects [50]. While the application of herbal antidotes in human poisoning cases remains untested, these findings warrant further investigation into potential adjunct therapies for oleander toxicity. Ceci et al. documented an outbreak of oleander poisoning in dairy cattle, emphasizing the risk of contamination in the food supply chain [3]. The study revealed clinical symptoms similar to those observed in humans, including gastrointestinal distress, severe bradycardia, and sudden cardiac failure. Necropsy findings highlighted myocardial degeneration, pulmonary congestion, and hepatic injury, reinforcing the cardiotoxic nature of oleandrin and its systemic impact across species [3].

### 3.7. Treatment of Oleander Poisoning

Immediate first aid measures are crucial in managing oleander poisoning to minimize toxin absorption and prevent severe complications [21,23,37,42,48,49,51,52,53]. When exposure is suspected, stopping further ingestion and removing any plant material from the mouth is a priority [21,23,40,42,48,49,51,52]. Rinsing the mouth with water and washing affected skin with soap and water can help reduce dermal absorption. Accidental ingestion, particularly in children, typically involves smaller doses and results in milder symptoms, whereas intentional ingestion—especially of seeds—leads to higher concentrations of cardiac glycosides and severe toxicity [23,42,48]. Inducing vomiting is not recommended due to aspiration risks; instead, activated charcoal can be administered to bind the toxins and limit systemic absorption [21,23,40,42,48,49,51,52]. These initial actions serve as a preliminary defense while awaiting medical assistance to stabilize the patient.

Medical interventions focus on supportive care, gastric decontamination, and antidotal therapy. Intravenous fluids are essential for maintaining hydration and supporting cardiovascular function, as cardiac glycosides compromise heart activity [52]. Continuous cardiac monitoring is critical, as common arrhythmias include bradycardia, atrioventricular (AV) block, and ventricular tachycardia—often the primary causes of mortality. Electrolyte imbalances, particularly hyperkalemia, occur due to Na^+^/K^+^-ATPase inhibition and require urgent correction with calcium gluconate, insulin with dextrose, sodium bicarbonate, or β-agonists, while hemodialysis may be necessary for refractory cases [27,51]. Bradycardia and AV block are typically managed with atropine, although in severe cases, temporary cardiac pacing may be required. Hypotension should be initially treated with intravenous fluids and, if necessary, vasopressors such as dopamine or norepinephrine. Digoxin-specific Fab antibody fragments (Digifab) are the most effective antidote, as they bind to cardiac glycosides and neutralize their effects, reversing poisoning [9,14,52]. However, high costs and limited availability in resource-constrained settings restrict their widespread use.

Gastric decontamination plays a crucial role in limiting absorption if the patient presents within a few hours of ingestion. Activated charcoal is the preferred method, administered as a single dose within 1–2 h of ingestion to bind oleandrin and reduce systemic absorption. Multiple doses may be considered in severe cases due to the enterohepatic recirculation of cardiac glycosides [21,23,40,42,48,49,51,52]. Gastric lavage is an option in large intentional ingestions but is generally reserved for severe cases due to the risks of aspiration and the availability of safer alternatives. Inducing emesis is discouraged, as vomiting occurs naturally due to oleandrin’s gastrointestinal irritant effects, and forced emesis can exacerbate bradycardia, aspiration risks, and electrolyte imbalances.

Long-term management involves careful monitoring and follow-up to ensure full recovery and prevent complications. Survivors may experience persistent cardiac, hepatic, or renal dysfunction, requiring ongoing evaluation for arrhythmias and organ damage [27]. In cases of intentional poisoning, psychological assessment and counseling are crucial to addressing underlying mental health concerns and preventing recurrence [4,23]. Nutritional support and the gradual reintroduction of normal dietary intake are important as the body recovers. Rehabilitation programs focusing on public education about oleander toxicity are essential, particularly in regions where the plant is widespread [5,14].

**Figure 1:**
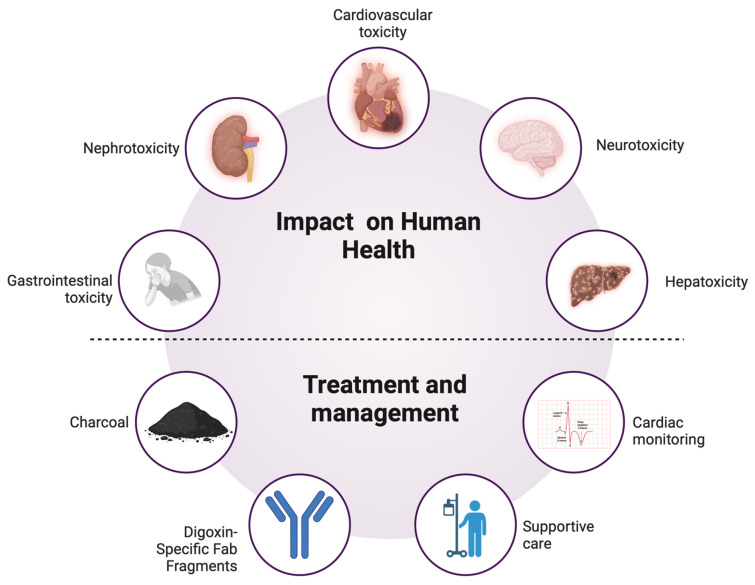
Impact of oleander poisoning on human health and treatment strategies.

Efforts to develop new antidotes and targeted therapies remain challenging. While research on digoxin-specific Fab fragments has demonstrated efficacy, its limited accessibility underscores the need for alternative treatments [52]. Autopsy findings provide critical insights into oleander toxicity, with myocardial necrosis, fibrosis, and varying cardiac lesions depending on dose, exposure duration, and survival time [41]. Understanding these pathological effects can inform the development of more effective treatment protocols. Identifying novel therapeutic targets may pave the way for cost-effective and widely available interventions, particularly in low-resource settings (Figure 1).

### 3.8. Data Gaps in Autopsy Studies and Their Impact on Therapeutic Strategies

Autopsy studies on fatal oleander poisoning provide crucial forensic insights, yet significant gaps remain in understanding the pathological mechanisms that could inform therapeutic strategies. Current postmortem reports often lack detailed histopathological assessments, making it difficult to correlate myocardial damage with the dose and timing of exposure. Unlike ischemic heart disease, where structural changes correspond to functional deficits, acute cardiac glycoside toxicity primarily affects biochemical and electrophysiological pathways, with histological alterations appearing only in cases of prolonged exposure or delayed fatalities. High-dose, rapid-onset poisoning may result in subtle myocardial swelling and hypereosinophilia, while prolonged survival can lead to myocardial necrosis, fibrosis, and inflammatory infiltration. Establishing a standardized approach to postmortem cardiac evaluation is essential to improve diagnostic accuracy and differentiate oleander toxicity from other causes of sudden cardiac death.

Experimental animal models offer a controlled setting to explore these pathological changes, addressing the limitations of human autopsy studies. Animal research has provided valuable insights into dose–response relationships, early biochemical markers of toxicity, and potential therapeutic interventions. Studies suggest that troponin release, mitochondrial dysfunction, and oxidative stress occur before structural myocardial damage becomes evident, underscoring the importance of early intervention.

### 3.9. Recommendations for Future Research and Policy

To increase the number of autopsies in cases of oleander poisoning, healthcare systems must implement targeted strategies that address both logistical and educational barriers. One effective approach could be increasing awareness among medical professionals about the importance of autopsies in understanding the specific mechanisms of death related to oleander toxicity. This awareness can be fostered through dedicated training sessions and workshops that highlight how autopsy findings can contribute to developing antidotes and improving clinical outcomes [44]. Additionally, establishing clear protocols for conducting autopsies in suspected poisoning cases can help streamline the process and ensure that valuable information is not lost. Implementing these strategies can lead to a more comprehensive collection of data, which is crucial for advancing research in this field.

Interdisciplinary collaboration is essential in enhancing research efforts related to oleander poisoning. By bringing together toxicologists, pathologists, botanists, and clinicians, a more holistic understanding of the toxic effects and potential treatment options can be achieved. For instance, toxicologists can provide insights into the chemical components of oleander that cause harm, while pathologists can interpret autopsy findings to determine the physiological impacts [39]. Policy recommendations are crucial to support autopsy-based research and improve clinical outcomes in oleander poisoning cases. Governments and health organizations should consider implementing policies that mandate autopsies in all suspected poisoning cases to ensure consistent data collection [51]. By establishing these policies, there can be a more structured approach to studying oleander poisoning, ultimately leading to better preventative measures and treatment options for affected individuals.

## 4. Conclusions

Oleander poisoning remains a significant clinical and forensic challenge. Despite advancements in diagnostic tools and treatment options, mortality remains a concern, especially in cases of intentional ingestion or delayed medical intervention. Fatal cases are often associated with severe cardiac toxicity, primarily bradycardia, high-grade atrioventricular blocks, and ventricular dysrhythmias, compounded by life-threatening hyperkalemia (>6.5 mmol/L). Autopsy studies consistently reveal myocardial congestion, necrosis, and systemic organ damage, reinforcing the need for standardizing forensic toxicology protocols to improve the accuracy of postmortem diagnosis.

While non-fatal cases are frequently reported, survivors often experience persistent cardiac, hepatic, or renal dysfunction, necessitating prolonged medical care. Epidemiological data indicate that suicidal ingestion accounts for a large proportion of cases, particularly in South Asia, where *Thevetia peruviana* poisoning is widespread. However, many suicide attempts involving oleander ingestion do not result in immediate death but lead to prolonged suffering, significant healthcare costs, and long-term complications. The limited availability of digoxin-specific Fab fragments in resource-poor settings remains a critical barrier to optimal management, highlighting the need for alternative, accessible antidotes and standardized treatment protocols.

Greater public awareness of the risks associated with oleander ingestion, coupled with improved mental health support and access to timely medical care, is crucial to reducing both accidental and intentional poisonings. Future research should prioritize comprehensive postmortem investigations to establish dose–response relationships and refine diagnostic criteria, particularly regarding subtle myocardial lesions, fibrosis, and electrolyte imbalances. Additionally, efforts to develop cost-effective therapies, enhance early detection strategies, and implement targeted prevention campaigns in high-risk areas could significantly reduce the global burden of oleander toxicity. Addressing these challenges will not only improve clinical and forensic outcomes but also mitigate the broader societal and healthcare impacts of oleander poisoning.

## 5. Materials and Methods

A comprehensive narrative review of the literature was conducted using the scientific database PubMed NCBI, covering studies published between January 2000 and January 2024. The search was performed using the primary keywords “oleander poisoning AND death”, with an initial screening of paper titles followed by a detailed review of abstracts to identify relevant studies. Papers that met the preliminary inclusion criteria were then subjected to a full-text analysis to ensure their relevance to the study objectives.

This review focused specifically on fatal cases of oleander poisoning in humans, aiming to consolidate knowledge on the epidemiology, clinical presentation, forensic findings, and treatment approaches associated with such cases. Studies that primarily addressed general toxicology, in vitro experiments, or poisonings caused by other cardiotoxic plants, such as *Cerbera odollam* and *Digitalis* species, were excluded unless they provided significant forensic insights applicable to human oleander toxicity. While forensic case reports formed the core of the review, broader clinical and epidemiological studies were also included to provide context regarding mortality trends and the role of medical intervention in patient outcomes.

In addressing the inclusion of animal studies, particular attention was given to their relevance in understanding oleander toxicity in humans. Only those studies that offered meaningful insights into the mechanisms of oleander-induced toxicity applicable to human pathophysiology were considered. These included both spontaneous cases of oleander poisoning in veterinary species, such as livestock and companion animals, as well as controlled experimental studies where oleander toxins were administered to animal models to investigate dose–response relationships and systemic effects. This review sought to differentiate between naturally occurring cases of oleander poisoning in animals, which may share some toxicological similarities with human exposures, and laboratory-based studies that provided controlled assessments of oleander’s impact on organ function, particularly the cardiovascular system.

The parameters analyzed in the selected forensic studies included demographic characteristics such as the age, gender, and geographical location of victims, as well as the type of exposure, whether accidental, deliberate, or occupational. Toxicological aspects, including the identification of *Nerium oleander* or *Thevetia peruviana* in postmortem samples, were evaluated in conjunction with clinical data detailing the time interval between ingestion and fatal intoxication, as well as documented symptoms preceding death. Forensic findings, particularly autopsy observations, histopathological changes, and toxicological confirmation, were carefully examined to understand the extent of organ involvement and systemic toxicity.

To maintain the quality and relevance of the data, certain exclusion criteria were applied. Non-English-language papers were not included in this review to ensure accessibility and standardization of data interpretation. Studies focused exclusively on in vitro experiments were omitted unless they provided direct forensic applications relevant to human poisoning cases. While experimental research on animal models was incorporated when it offered valuable insights into the pathophysiology of fatal oleander intoxication, veterinary case reports unrelated to human exposures were excluded to maintain the focus on human morbidity and mortality.

By integrating forensic case reports, clinical studies, and epidemiological data, this review aimed to provide a multidisciplinary perspective on fatalities associated with oleander poisoning. The findings highlight the complex interplay between clinical presentation, toxicological confirmation, and forensic investigations in cases of fatal oleander poisoning, underscoring the need for standardized diagnostic and therapeutic approaches to improve patient outcomes and forensic accuracy.

## Figures and Tables

**Table 1:** Results of literature review.

Author and Year	Study Type	Location and Population	Study Focus and Methodology	Clinical Findings	Outcomes and Key Insights
Krishnasamy et al. (2024) [28]	Retrospective study	South India, 2123 cases analyzed	Investigated the epidemiology of oleander poisoning in a tertiary care hospital. Focused on demographics, clinical presentation, and mortality predictors.	Most cases involved young rural males; GCS score was identified as a strong predictor of mortality. Symptoms included vomiting, abdominal pain, and cardiac dysrhythmias.	Lower GCS scores were associated with poorer outcomes, highlighting the need for rapid intervention and early identification of high-risk patients.
Ayyappan et al. (2023) [29]	Case report	India, pediatric case	Described a fatal case of accidental ingestion of Nerium oleander leaves in a child. Autopsy findings were analyzed to identify systemic effects.	Severe symptoms included vomiting, hypotension, hyperkalemia, and myocardial dysfunction. Death occurred within 36 h.	Demonstrates the systemic toxicity of oleander in children, underscoring the importance of timely diagnosis and the role of autopsy in determining cause of death.
Torrents et al. (2023) [30]	Regional analysis	Southeastern France, 20-year review	Reviewed 71% of plant-related poisonings attributed to oleander. Focused on cases of deliberate ingestion and the associated clinical and forensic outcomes.	Documented multi-organ congestion and cardiac failure in fatal cases. Severe cardiac dysrhythmias were common.	Emphasizes the lack of standardized treatment protocols, suggesting the need for consistent therapeutic guidelines.
Subramani et al. (2023) [1]	Observational study	India, yellow oleander cases	Examined clinical and biochemical changes in patients with yellow oleander poisoning. Focused on electrolyte disturbances and cardiac manifestations.	Persistent vomiting, hyperkalemia, bradycardia, AV blocks, and ST-segment changes on ECG. Crushed seeds caused more severe symptoms.	Serum potassium levels were a critical predictor of cardiotoxicity. Reinforces the importance of early electrolyte correction and cardiac monitoring.
Carfora et al. (2021) [31]	Forensic case report	Italy, suicide case	Described a suicide case involving the ingestion of a Nerium oleander infusion. Toxicological analysis confirmed oleandrin presence.	High oleandrin levels were found in blood and tissue samples. Cause of death was fatal cardiac arrhythmia.	Highlights the utility of advanced toxicological methods like LC-MS/MS in identifying oleander toxins in forensic investigations.
Papi et al. (2012) [32]	Forensic case report	Italy, double fatality	Investigated a rare double fatality from accidental ingestion of oleander leaves. Autopsy and toxicological analyses confirmed cardiac glycosides.	Fatalities resulted from cardiac failure. Toxicological tests confirmed high levels of cardiac glycosides.	Stresses the importance of thorough toxicological analysis in distinguishing oleander poisoning from other causes of sudden death.
Roberts et al. (2016) [33]	Review	Global	Comprehensive review of treatment strategies for cardiac glycoside poisoning. Focused on various interventions.	Recommended interventions included activated charcoal, atropine, temporary cardiac pacing, and Fab fragments.	Warns about the financial and logistical constraints of Fab fragments, advocating for affordable therapies.
Bandara et al. (2010) [23]	Review	South Asia	Reviewed clinical management and diagnosis of Nerium oleander and Thevetia peruviana poisoning. Emphasized supportive care.	Key manifestations included gastrointestinal symptoms, cardiac dysrhythmias, and hyperkalemia.	Reinforces the importance of supportive care and Fab fragments for severe poisoning.
Gawarammana et al. (2010) [34]	Randomized controlled trial	Sri Lanka, yellow oleander cases	Investigated the efficacy of Fructose 1,6-diphosphate (FDP) as a novel antidote for cardiac toxicity.	FDP showed potential in reversing life-threatening arrhythmias and improving survival rates.	Suggests FDP as a cost-effective alternative to Fab fragments in resource-limited areas.
Carroll et al. (2012) [35]	Epidemiological study	United Kingdom	Explored the influence of ingestion timing on outcomes in oleander poisoning.	Ingestion during the evening was associated with a lower case fatality rate.	Provides evidence of chronotoxicity, suggesting circadian variations in metabolism may affect outcomes.
Anandhi et al. (2018) [11]	Case report	India, acute myocardial infarction case	Documented a rare case of myocardial infarction following yellow oleander poisoning.	Patient initially had normal sinus rhythm but developed acute myocardial infarction.	Highlights the wide spectrum of cardiac complications and the importance of continuous cardiac monitoring.
Bose et al. (1999) [36]	Retrospective study	India, 300 cases reviewed	Analyzed 300 accidental yellow oleander poisoning cases, identifying patterns of morbidity and mortality.	Fourteen fatalities reported. Common autopsy findings included subendocardial hemorrhage and multi-organ involvement.	Provides valuable insights into the pathology of oleander poisoning, stressing the role of autopsy in understanding effects.

## Data Availability

No new data were created or analyzed in this study.
